# Supplementary material for: Molecular mechanism for vitamin C-derived C5-glyceryl-methylcytosine DNA modification catalyzed by algal TET homologue CMD1
Source: Nat Commun. 2021 Feb 2;12:744. doi: 10.1038/s41467-021-21061-2 (PMC7854593; doi:10.1038/s41467-021-21061-2)
Supplement: Supplementary file 1 — Supplementary Information [file 41467_2021_21061_MOESM1_ESM.pdf]

Supplementary Information

Molecular mechanism for vitamin C-derived C^5-glyceryl-methylcytosine DNA modification catalyzed by algal TET homologue CMD1

Wenjing Li[1,$], Tianlong Zhang[1,$], Mingliang Sun[1,$], Yu Shi[1,2], Xiao-Jie Zhang[1], Guo-Liang Xu[1], and Jianping Ding[1,2,3,*]

[1] State Key Laboratory of Molecular Biology, Shanghai Institute of Biochemistry and Cell Biology, Center for Excellence in Molecular Cell Science, Chinese Academy of Sciences; University of Chinese Academy of Sciences, 320 Yue-Yang Road, Shanghai 200031, China

[2] School of Life Science and Technology, ShanghaiTech University, 393 Hua-Xia Zhong Road, Shanghai 201210, China

[3] School of Life Science, Hangzhou Institute for Advanced Study, University of Chinese Academy of Sciences, 1 Xiangshan Road, Hangzhou 310024, China

* To whom correspondence should be addressed: Dr. Jianping Ding, Phone: 086-21-54921619, E-mail: jpding@sibcb.ac.cn.

[$] These authors contributed equally to this work.

Supplementary Table 1. Summary of the DNA substrates used in this study and the binding affinities of the full-length CMD1 towards these DNA substrates. The black sphere represents the 5'-biotin of the DNA. The binding affinities of CMD1 with different DNAs were analyzed by the bio-layer interferometry assay (see Methods).

DNA	Structure	Sequence	Length	K_D (μM)
14-nt DNA	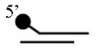	5'-biotin-CCCGCGCGGGATGT-3'<br>TGTAGGGCGCGCCC-5'	14 nt	2.01 ± 0.11
dsDNA1	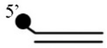	5'-biotin-GCTTTAACTTACATAAATGCCAATATAGTATGCGTATTGC-3'<br>CGAAATTGAATGTATTTACGGTTATATCATACGCATAACG-5'	40 bp	2.50 ± 0.15
dsDNA2	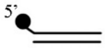	5'-biotin-ATACTTACGAATCTGAACTTAGATCCTAATATACATTCTA-3'<br>TATGAATGCTTAGACTTGAATCTAGGATTATATGTAAGAT-5'	40 bp	2.09 ± 0.13
dsDNA3	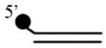	5'-biotin-GCTGGCCCTGACATGGGTGCCAGCGTAGTGCGCGTAGCGC-3'<br>CGACCGGGACTGTACCCACGGTCGCATCACGCGCATCGCG-5'	40 bp	2.03 ± 0.12
dsDNA4	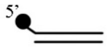	5'-biotin-GACATCGGCGCGGGGTTCAGCCCCGGCCGTATTGGGGCTC-3'<br>CTGTAGCCGCGCCCCAAGTCGGGGCCGGCATAACCCCGAG-5'	40 bp	1.97 ± 0.12
dsDNA5	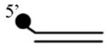	5'-biotin-GCTTTAACTTACATAAATGC(^{m}C)AATATAGTATGCGTATTGC-3'<br>CGAAATTGAATGTATTTACG G TTATATCATACGCATAACG-5'	40 bp	2.28 ± 0.14
dsDNA6	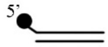	5'-biotin-GCTTTAA(^{m}C)TTACATAAATGC(^{m}C)AATATAGTATGCGTATTGC-3'<br>CGAAATT G AATGTATTTACG G TTATATCATACGCATAACG-5'	40 bp	2.52 ± 0.16
dsDNA7	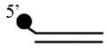	5'-biotin-TTGCCAGTAT-3'<br>AACGGTCATA-5'	10 bp	2.14 ± 0.11
dsDNA8	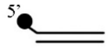	5'-biotin-ACTTACTGCCAATATAGTAT-3'<br>TGAATGACGGTTATATCATA-5'	20 bp	1.74 ± 0.10
dsDNA9	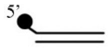	5'-biotin-AACTTACATAAATGCCAATATAGTATGCGT-3'<br>TTGAATGTATTTACGGTTATATCATACGCA-5'	30 bp	2.15 ± 0.13
ssDNA	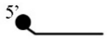	5'-biotin-TTTTTTCTTTTTTCTTTTTTCTTTTTTCTTTTTTCTTTTT-3'	40 nt	2.77 ± 0.17
Y-DNA	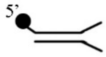	TATGCGTATTGC-3'<br>5'-biotin-GCTTTAACTTACATAAATGCCAATATAG<br>CGAAATTGAATGTATTTACGGTTATATC<br>CGCATACGCCAT-5'	28 bp<br>+<br>12 nt	1.41 ± 0.08
Bubble	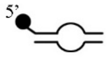	CCCCCCCCCCCC<br>5'-biotin-GCTTTAACTTACATA TAGTATGCGTATTGC-3'<br>CGAAATTGAATGTAT ATCATACGCATAACG-5'<br>TTTTTTTTTTTT	14 bp<br>+<br>12 nt<br>+<br>14 bp	1.19 ± 0.08
5'-Flap	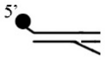	5'-biotin-GCTTTAACTTACATAAATGCCAATATAG —TATGCGTATTGC-3'<br>CGAAATTGAATGTATTTACGGTTATATC ATACGCATAACG-5'<br>CGCATACGCCAT-5'	40 bp<br>+<br>12 nt	1.35 ± 0.09
3'-Flap	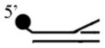	TATGCGTATTGC-3'<br>5'-biotin-GCTTTAACTTACATAAATGCCAATATAG GCGTATGCGGTA-3'<br>CGAAATTGAATGTATTTACGGTTATATC —CGCATACGCCAT-5'	40 bp<br>+<br>12 nt	1.34 ± 0.09
5'-OH	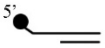	5'-biotin-GCTTTAACTTACATAAATGCCAATATAGTATGCGTATTGC-3'<br>CGGTTATATCATACGCATAACG-5'	18 nt<br>+<br>22 bp	1.61 ± 0.10
3'-OH	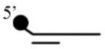	5'-biotin-GCTTTAACTTACATAAATGCCAATATAGTATGCGTATTGC-3'<br>CGAAATTGAATGTATTTA-5'	18 bp<br>+<br>22 nt	1.26 ± 0.08

Supplementary Table 2. Summary of the 90-bp dsDNAs containing six 5mCpX sites, where X is G, A, C, or T, used in this study. The positions of methylated cytosine (5mC) are indicated as ^{m}C.

DNA	Sequence
CpA	5'-AATGGCACTGCC(^{m}C)ACTTGGTGCAGTACTTACGCAAGTGATTAT(^{m}C)ACCTTTATGCCAAGTCCCGACGT(^{m}C)AATGACGAAACCGCCTGGCATT-3'<br>3'-TTACCGTGACGGGTGAA(^{m}C)CACGTCATGAATGCGTTCA(^{m}C)TAATAGTGGAAATACGGTTCAGGGCTGCAGTTA(^{m}C)TGCTTTGGCGGACCGTAA-5'
CpT	5'-AATGGCACTGCCCA(^{m}C)TTGGTGCAGTACTTACGCAAGTGATTATCAC(^{m}C)TTTATGCCAAGTCCCGACGTCAATGACGAAACCGC(^{m}C)TGGCATT-3'<br>3'-TTACCGTGACGGGTGAACCACGT(^{m}C)ATGAATGCGTT(^{m}C)ACTAATAGTGGAAATACGGTT(^{m}C)AGGGCTGCAGTTACTGCTTTGGCGGACCGTAA-5'
CpC	5'-AATGGCACTGC(^{m}C)CACTTGGTGCAGGCAAGTGATTATCA(^{m}C)CTTTATGCCAAGTGGCGACGTCAATGACGAAA(^{m}C)CGCCTGGCATTACTTACT-3'<br>3'-TTACCGTGACGGGTGAAC(^{m}C)ACGTCCGTTCACTAATAGTGGAAATACGGTTCAC(^{m}C)GCTGCAGTTACTGCTTTGGCGGAC(^{m}C)GTAATGAATGA-5'
CpG	5'-AATGACACTGCC(^{m}C)GCACTGTGGTGCAGGCGAGTCATTAT(^{m}C)GACTTTATGCCAAGTGACGTCAATGA(^{m}C)GAATTACTTAACCGCCTGGCACT-3'<br>3'-TTACTGTGACGGGCGTGACACCACGTCCG(^{m}C)TCAGTAATAGCTGAAATACGGTTCACTG(^{m}C)AGTTACTGCTTAATGAATTGG(^{m}C)GGACCGTGA-5'

Supplementary Table 3. Summary of the defined regions of MBP and CMD1 and nucleotides of DNA in different CMD1 structures.

		Apo	VC	5mC-DNA	DNA	5mC-DNA+VC
MBP (1-367)		8-54,<br>58-367	5-367	3-367	3-367	8-53, 59-367
CMD1 (1-532)		1-176,<br>185-243,<br>250-508	1-175,<br>185-242,<br>250-508	1-176,<br>185-508	1-176,<br>184-507	1-176, 185-508
DNA	Substrate strand	-	-	1-7	1-7	1-8
	Non-substrate strand	-	-	3-11	3-11	3-11

Supplementary Table 4. The types and abundances of metal ions in the protein solutions as analyzed by inductively coupled plasma optical emission spectrometer (ICP-OES) using Leeman Prodigy. The concentration (%) of detected metal ions in the CMD1 protein solution without [Fe (-)] or with [Fe (+)] supplementation of $(NH_4)_2Fe(SO_4)_2$ are shown.

Metal	Concentration (%)	
	Fe (-)	Fe (+)
Fe	0.045	0.078
Ca	0.032	0.010
Ni	0.009	0.019

Supplementary Table 5. Comparison of CMD1 in different structures.

		VC	5mC-DNA	DNA	5mC-DNA+VC
Apo	RMSD (Å)	0.12	0.24	0.23	0.29
	Aligned Cα atoms	492	494	493	493

Supplementary Table 6. Summary of primers used in this study.

Primer	Sequence
CMD1-aa1-NotI-F	5’-ATAAGAATGCGGCCGCAATGAGTGTCGCCCTAGCATCG-3’
CMD1-aa509-NotI-R	5’-ATAAGAATGCGGCCGCAGCCGGAGGCTTCCGCCAG-3’
CMD1-aa532-NotI-R	5’-ATAAGAATGCGGCCGCTGCGCCACAAAAATCGACAT-3’
DNA-Sub-F	5’-biotin-AAACTGCCCACTTGGCAGTACATCAAGT-3’
DNA-Sub-R	5’-CAGAATTCCACCACACTGGACTAGTGGA-3’

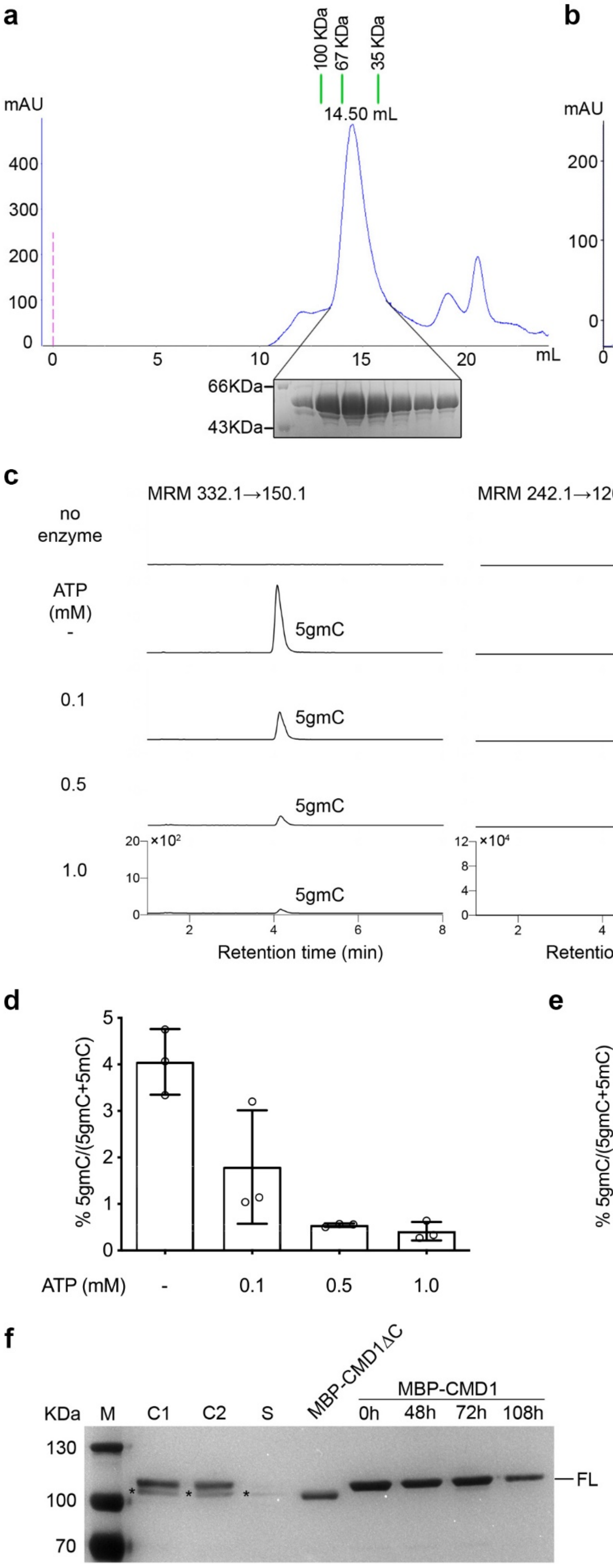
a
100 KDa
67 KDa
35 KDa
mAU
14.50 mL
400
300
200
100
0
0
5
10
15
20
mL
66KDa
43KDa
b
100 KDa
67 KDa
35 KDa
mAU
13.32 mL
200
100
0
0
4
8
12
16
20
mL
130KDa
100KDa
c
MRM 332.1→150.1
MRM 242.1→126.1
no enzyme
ATP (mM)
-
0.1
0.5
1.0
5gmC
5mC
20
10
0
×10²
12
8
4
0
×10⁴
2
4
6
8
Retention time (min)
Retention time (min)
d
% 5gmC/(5gmC+5mC)
5
4
3
2
1
0
ATP (mM)
-
0.1
0.5
1.0
e
% 5gmC/(5gmC+5mC)
4
3
2
1
0
5mCpG DNA
5mCpC DNA
5mCpA DNA
5mCpT DNA
f
KDa
M
C1
C2
S
MBP-CMD1ΔC
MBP-CMD1
0h
48h
72h
108h
130
100
70
FL

Supplementary Fig. 1. Purification and biochemical characterization of CMD1. (a) Size-exclusion chromatography (SEC, G200 10/300) and SDS-PAGE (10%) analysis results of the full-length wild-type CMD1 (residues 1-532) used in the biochemical studies. The standard molecular markers are indicated. (b) Size-exclusion chromatography (G200 10/300) and SDS-PAGE (10%) analysis results of the MBP-fused full-length CMD1 used in the structural studies. Uncropped gels are provided in Source Data. (c) Enzymatic activities of the wild-type CMD1 in the absence and presence of ATP analyzed with the LC/MS MRM detection. Representative LC/MS chromatograms in MRM acquisition mode are shown. Selected ions for nucleosides were as follows: MRM transitions 228.1→112.1, 242.1→126.1, 258.1→142.1, 272.1→156.1, and 332.1→150.1 for the detection of C, 5mC, 5hmC, 5caC, and 5gmC, respectively. (d) Enzymatic activities of the wild-type CMD1 in the presence and absence of ATP. The enzymatic activity of CMD1 is represented by the ratio of the 5gmC over the total of the 5gmC and the remaining 5mC. Data are presented as mean values ± SEM (standard error of the mean). n = 3 independent replicates. (e) Enzymatic activities of the wild-type CMD1 towards 90-bp dsDNAs containing six 5mCpX sites (X = G, A, C, or T). Data are presented as mean values ± SEM. n = 3 independent replicates. (f) Silver staining of SDS-PAGE (7%) analysis results of protein stability of the MBP-fused CMD1 in the storage buffer, and the crystals (C1 and C2) and the crystallization solution (S) of the MBP-fused CMD1. The degraded band of CMD1 is indicated with an asterisk. For comparison, the C-terminal truncated MBP-fused CMD1 (CMD1ΔC, residues 1-509) was also included. This experiment was repeated thrice independently with similar results. Uncropped gels are provided in Source Data.

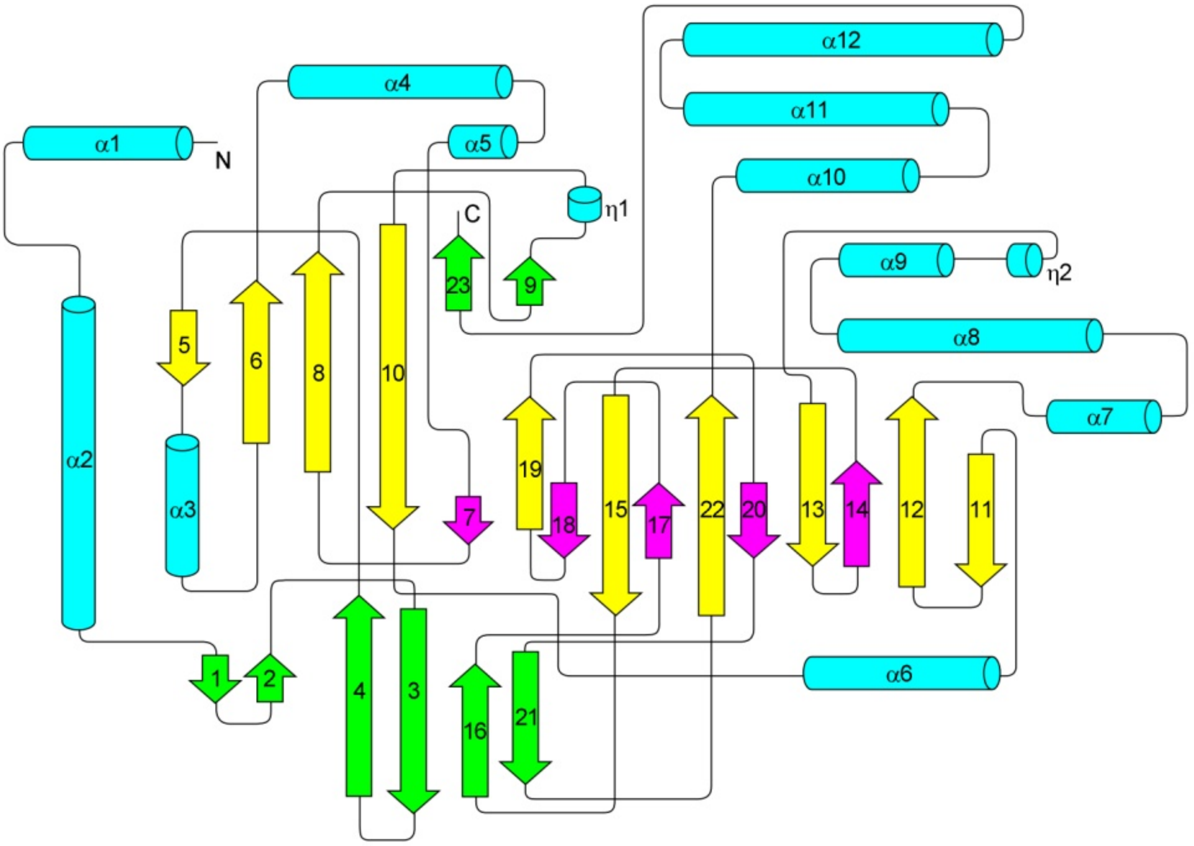

Supplementary Fig. 2. Topology diagram of CMD1. The minor β-sheet and major β-sheet of the DSBH fold are colored in magenta and yellow, respectively. The two layers of α-helices are colored in cyan. The extra β-strands flanking the DSBH fold are colored in green. The secondary structure elements are labeled. The N- and C-termini are indicated.

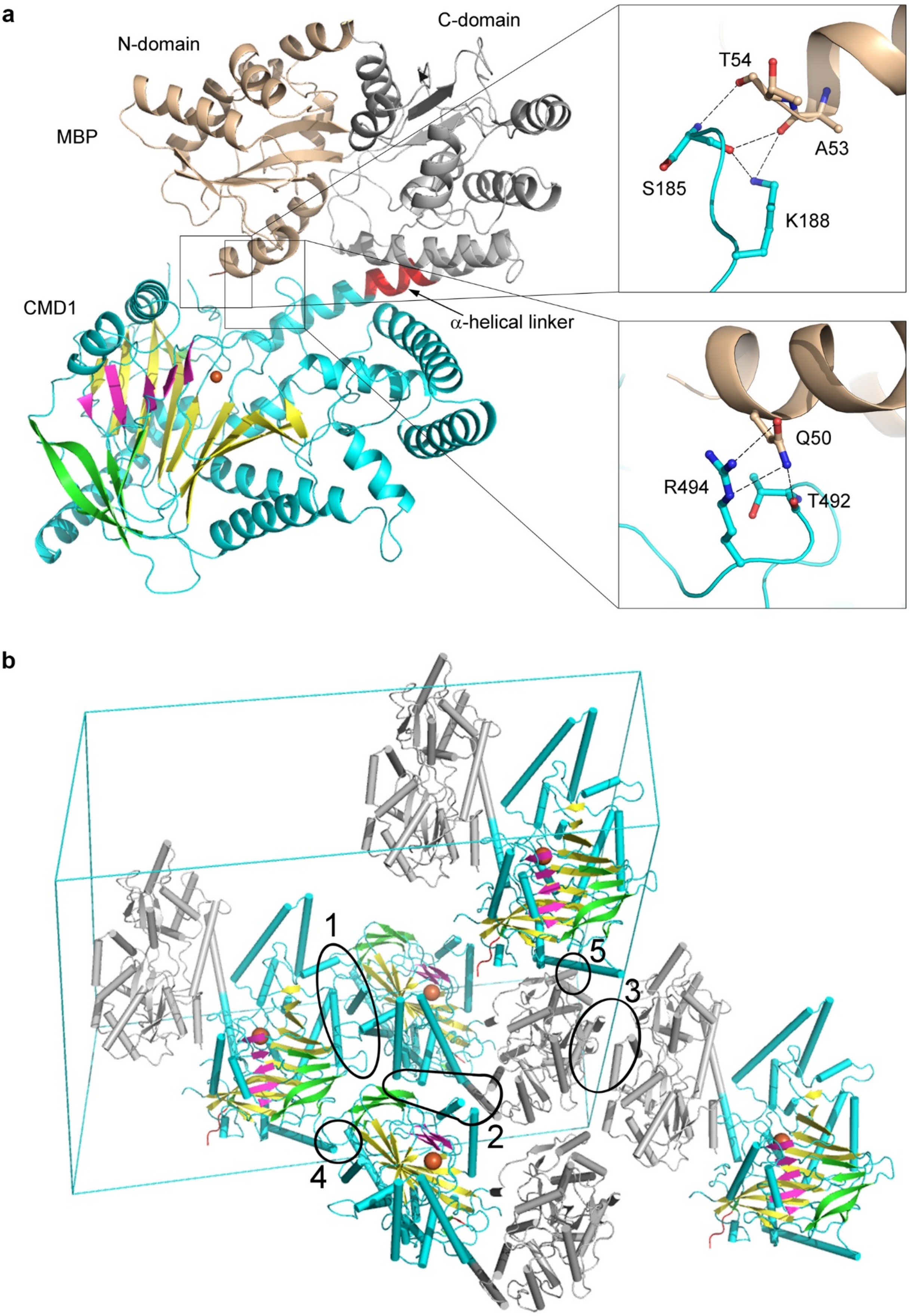
a
N-domain
C-domain
MBP
CMD1
α-helical linker
T54
A53
S185
K188
Q50
R494
T492
b
1
2
3
4
5

Supplementary Fig. 3. Overall structure of the MBP-fused CMD1 in apo form. (a) The MBP was fused to the N-terminus of CMD1 via an α-helical linker, resulting in the formation of a long α-helix comprising the last α-helix of MBP, the α-helical linker, and the α1 helix of CMD1. The MBP is located in adjacent to CMD1, and its position and orientation are dictated by the long α-helix. The N-domain and C-domain of MBP are shown in ribbon representation and colored in wheat and gray, respectively; and the α-helical linker between MBP and CMD1 is colored in red. The zoom-in windows show the hydrogen-bonding interactions between the N-domain of MBP and CMD1. The residues involved in the interactions are shown with ball-and-stick models. The hydrogen bonds are indicated with black dotted lines. (b) Crystal packing of the MBP-CMD1 structure in apo form. The CMD1 is colored as in Fig. 1a and the MBP is colored in gray. The location of the active site is indicated by the position of the bound Fe^{2+} which is shown with a red sphere. The unit cell box is shown in cyan. There is one MBP-CMD1 molecule in the asymmetric unit. The inter-molecular contacts at site 1 (the CMD1-CMD1 interactions), site 2 (the CMD1-MBP interactions), and site 3 (the MBP-MBP interactions) make the major contributions in the crystal packing. The inter-molecular contacts at site 4 (the CMD1-CMD1 interactions) and site 5 (the CMD1-MBP interactions) make the minor contributions in the crystal packing. The C-terminus of CMD1 is colored in red. The structure elements of CMD1 involved in the crystal packing (α2 and α5-α8 helices) are located distantly from the active site of CMD1, and thus the crystal packing should have no direct impact on the chemical property and structure of the active site. In addition, the MBP is also located distantly from the active site of CMD1, and thus should have no direct impact on the chemical property and structure of the active site as well.

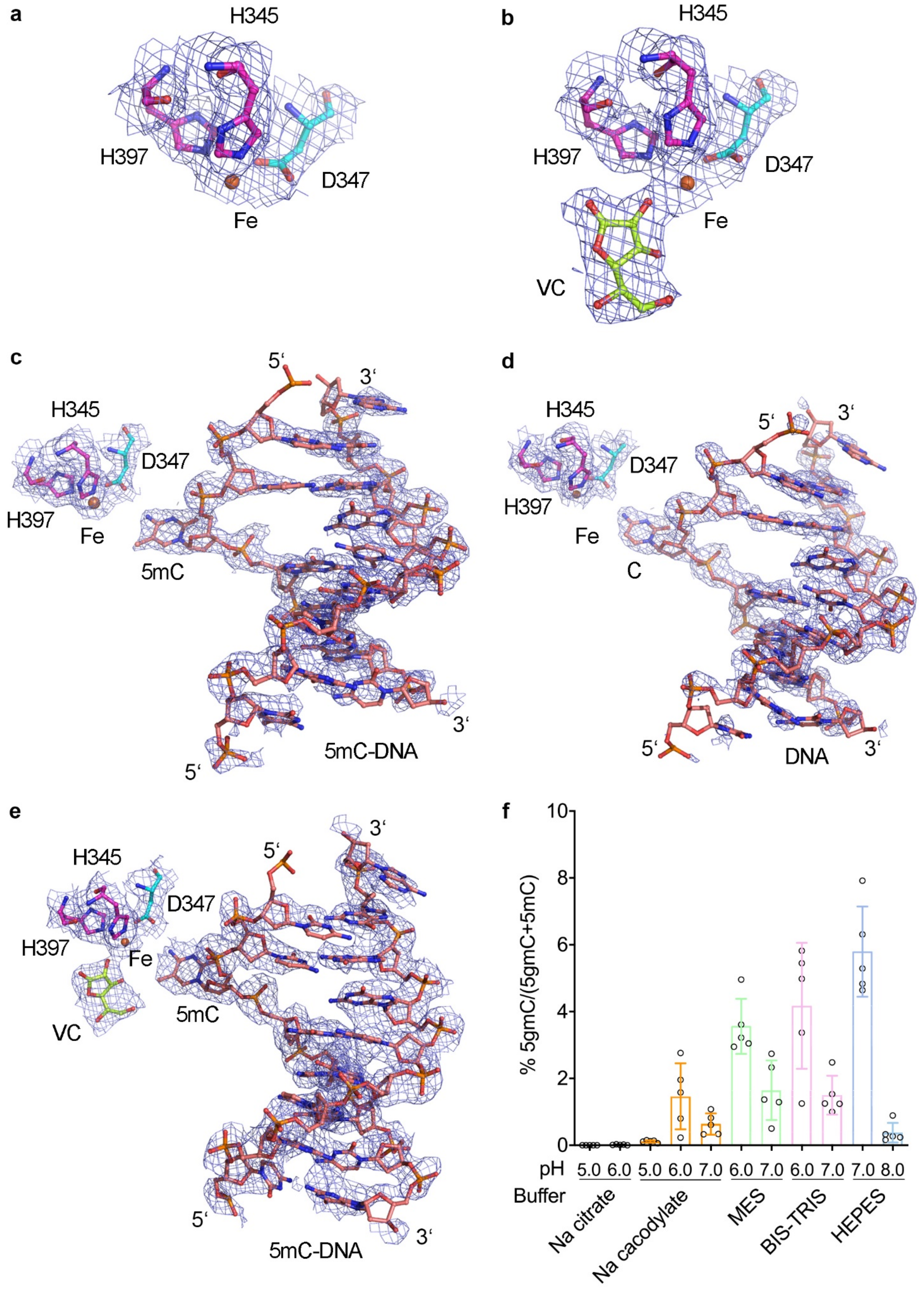
a
H345
H397
D347
Fe
b
H345
H397
D347
Fe
VC
c
5‘
3‘
H345
D347
H397
Fe
5mC
3‘
5‘
5mC-DNA
d
H345
5‘
3‘
D347
H397
Fe
C
5‘
DNA
3‘
e
3‘
5‘
H345
D347
H397
Fe
VC
5mC
5‘
3‘
5mC-DNA
f
% 5gmC/(5gmC+5mC)
10
8
6
4
2
0
pH
5.0 6.0 5.0 6.0 7.0 6.0 7.0 6.0 7.0 7.0 8.0
Buffer
Na citrate
Na cacodylate
MES
BIS-TRIS
HEPES

Supplementary Fig. 4. Representative simulated annealing composite omit maps (contoured at 1.0 σ level) in different CMD1 structures. (a) The active site in the apo CMD1. (b) The active site in the CMD1-VC complex. (c) The active site and the bound DNA in the CMD1-5mC-DNA complex. (d) The active site and the bound DNA in the CMD1-DNA complex. (e) The active site and the bound DNA in the CMD1-5mC-DNA-VC complex. The residues, nucleotides and VC are shown with ball-and-stick models. (f) Effects of different buffers with different pH values on the enzymatic activities of the wild-type CMD1. Data are presented as mean values ± SEM. n = 5 independent replicates.

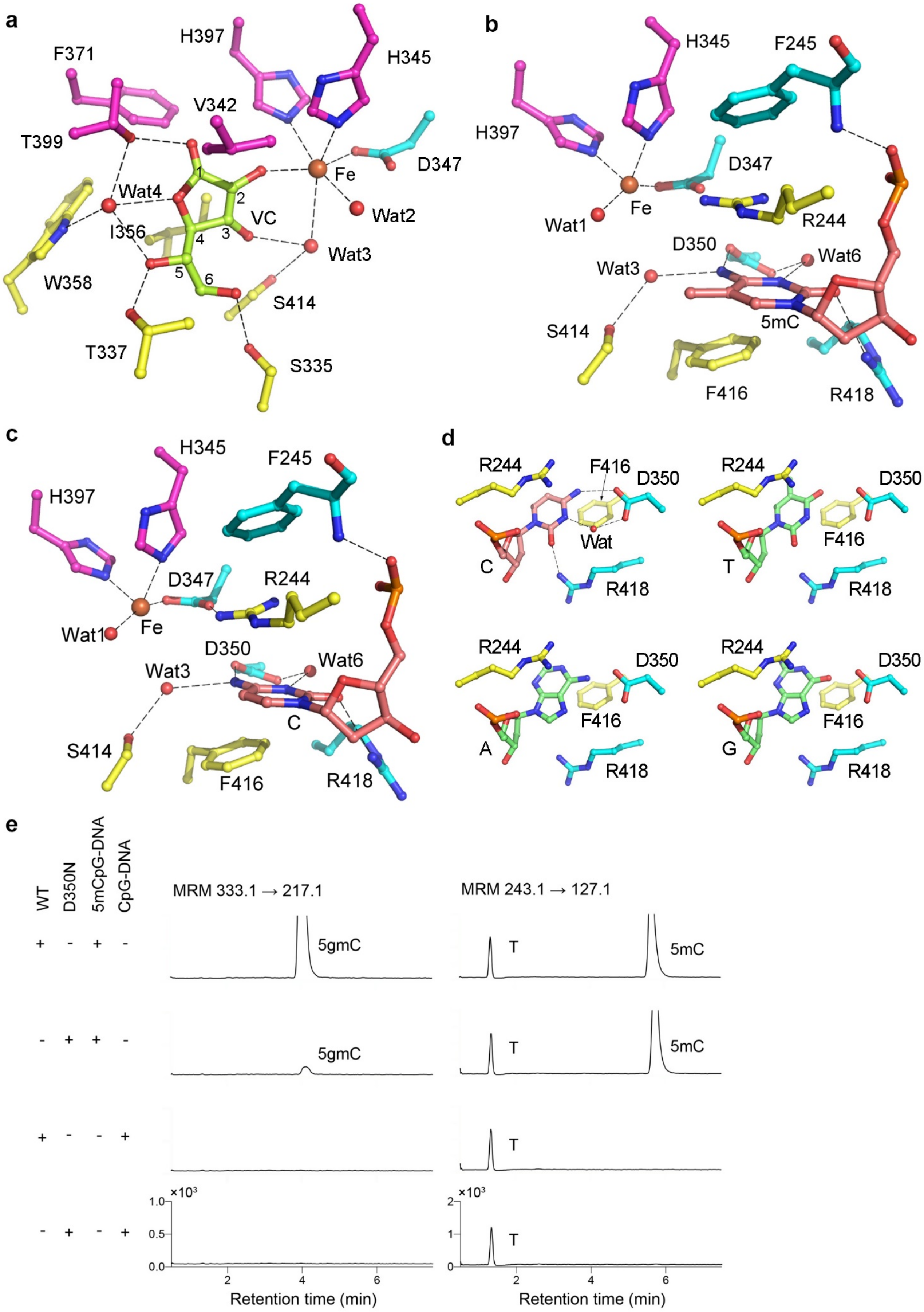

a
F371
H397
H345
V342
T399
Fe
D347
Wat4
Wat2
VC
I356
Wat3
W358
S414
T337
S335
b
H345
F245
H397
D347
Fe
Wat1
R244
D350
Wat6
Wat3
5mC
S414
F416
R418
c
H345
F245
H397
D347
R244
Fe
Wat1
D350
Wat6
Wat3
C
S414
F416
R418
d
R244
F416
D350
Wat
C
R418
T
A
G
e
WT
D350N
5mCpG-DNA
CpG-DNA
MRM 333.1 → 217.1
MRM 243.1 → 127.1
5gmC
T
5mC
×10³
Retention time (min)

Supplementary Fig. 5. Structure of the active site of CMD1. (a) Structure of the active site of CMD1 in complex with VC showing the interactions of the Fe^{2+} and the VC with the surrounding residues. (b-c) Structure of the active site of CMD1 in complex with 5mC-DNA (b) and with DNA (c) showing the interactions of the Fe^{2+} and the flipped-out 5mC/C base with the surrounding residues. The residues, nucleotides and VC are shown with ball-and-stick models. The Fe^{2+} and water molecules are shown in iron-red and red spheres, respectively. The color coding is same as Fig. 1b. (d) Docking studies of A, T, and G bases into the active site of CMD1 based on the location of the flipped-out C base. The residues involved in the recognition of the C base and the modeled bases are shown with ball-and stick models. The color coding for the residues and the flipped-out C base is the same as Fig. 1d. The modeled bases are colored in lime. (e) Enzymatic activities of the wild-type and D350N mutant CMD1 towards 0.5-kb 5mCpG-DNA or CpG-DNA analyzed with the LC/MS MRM detection. Representative LC/MS chromatograms in MRM acquisition mode are shown. Selected ions for nucleosides were as follows: MRM transitions 228.1→112.1, 242.1→126.1, 258.1→142.1, 272.1→156.1, 332.1→150.1, and 243.1→127.1 for the detection of C, 5mC, 5hmC, 5caC, 5gmC, and T, respectively. No new product peak could be identified at the expected position for C^5-glyceryl-thymine (5gT or 5gmU) (333.1→217.1).

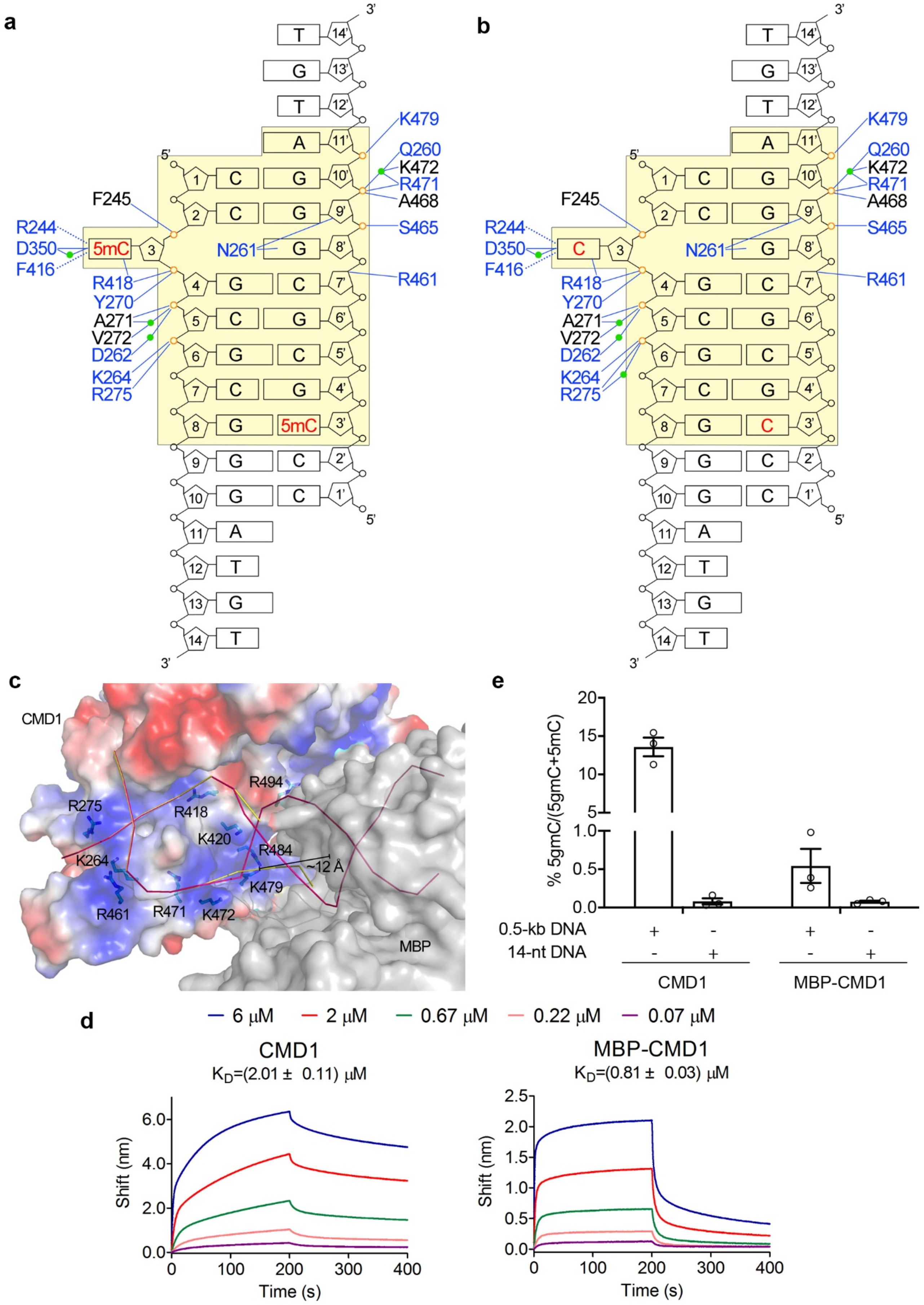
a
b
5'
3'
R244
D350
F416
5mC
F245
N261
R418
Y270
A271
V272
D262
K264
R275
K479
Q260
K472
R471
A468
S465
R461
C
c
CMD1
R275
R418
R494
K420
R484
K264
~12 Å
K479
R461
R471
K472
MBP
e
% 5gmC/(5gmC+5mC)
0.5-kb DNA
14-nt DNA
CMD1
MBP-CMD1
d
6 μM
2 μM
0.67 μM
0.22 μM
0.07 μM
CMD1
K_D=(2.01 ± 0.11) μM
MBP-CMD1
K_D=(0.81 ± 0.03) μM
Shift (nm)
Time (s)

Supplementary Fig. 6. Interactions between CMD1 and DNA. (a-b) Schematic representations of the interactions between CMD1 and the methylated DNA (a) or unmethylated DNA (b). The defined region of the bound DNA in the crystal structure is highlighted in a shaded yellow box. The hydrophilic interactions are indicated with blue solid lines and the hydrophobic contacts or cation/π-π stacking interactions with blue dashed lines. The phosphates involved in the interactions with CMD1 are highlighted in orange. The residues interacting with the bound DNA via the side chains and main chains are colored in blue and black, respectively. Water molecules are indicated with green spheres. (c) Modeling studies of CMD1 with longer DNAs. The disordered 3'-overhang of the non-substrate strand of the bound DNA in the CMD1-5mC-DNA-VC complex is modeled based on the extension of the defined region of the non-substrate strand. In addition, a 20-bp dsDNA was also modeled based on the extension of the dsDNA region of the bound DNA in the CMD1-5mC-DNA-VC complex. The modeled 14-nt dsDNA and 20-bp dsDNA are shown in ribbon and colored in yellow and magenta, respectively. The CMD1 is shown as electrostatic potential surface and the MBP is shown as gray surface. The basic residues located at or in adjacent to the DNA-binding site of CMD1 are shown with ball-and-stick models. (d) Binding affinities of CMD1 and MBP-CMD1 towards the 14-nt 5mC-DNA used in the structural studies. The K_D value was indicated on the top of the corresponding BLI sensorgram. The concentrations of the wild-type CMD1 used in the experiments are indicated on the top of the figure. (e) Enzymatic activities of CMD1 and MBP-CMD1 towards 0.5-kb DNA used in the biochemical studies and the 14-nt 5mC-DNA used in the structural studies. Data are presented as mean values ± SEM. n = 3 independent replicates.

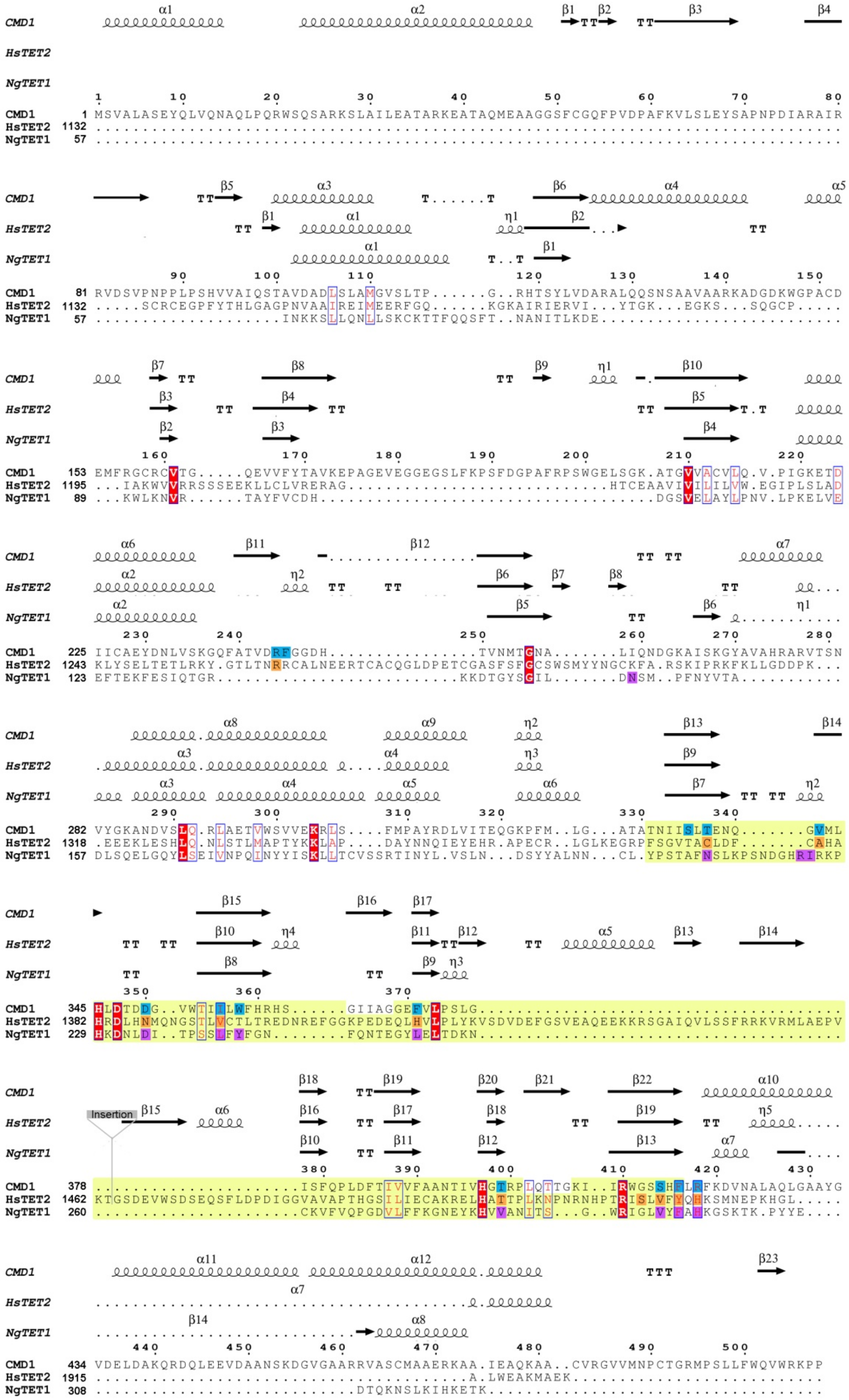

CMD1
HsTET2
NgTET1
Insertion
CMD1 1 MSVALASEYQLVQNAQLPQRWSQSARKSLAILEATARKEATAQMEAAGGSFCGQFPVDPAFKVLSLEYSAPNPDIARAIR
HsTET2 1132
NgTET1 57
CMD1 81 RVDSVPNPPLPSHVVAIQSTAVDADLSLAMGVSLTP......G..RHTSYLVDARALQQSNSAAVAARKADGDKWGPACD
HsTET2 1132 .....SCRCEGPFYTHLGAGPNVAAIREIMEERFGQ......KGKAIRIERVI...YTGK...EGKS...SQGCP.....
NgTET1 57 ....................INKKSLLQNLLSKCKTTFQQSFT..NANITLKDE....................
CMD1 153 EMFRGCRCVTG.....QEVVFYTAVKEPAGEVEGGEGSLFKPSFDGPAFRPSWGELSGK.ATGVVACVLQ.V.PIGKETD
HsTET2 1195 ...IAKWVVRRSSSEEKLLCLVRERAG.......................HTCEAAVIVILILVW.EGIPLSLAD
NgTET1 89 ...KWLKNVR......TAYFVCDH................................DGSVELAYLPNV.LPKELVE
CMD1 225 IICAEYDNLVSKGQFATVDRFGGDH................TVNMTGNA.......LIQNDGKAISKGYAVAHRARVTSN
HsTET2 1243 KLYSELTETLRKY.GTLTNRRCALNEERTCACQGLDPETCGASFSFGCSWSMYYNGCKFA.RSKIPRKFKLLGDDPK...
NgTET1 123 EFTEKFESIQTGR.......................KKDTGYSGIL.......DNSM..PFNYVTA..........
CMD1 282 VYGKANDVSLQ.RLAETVWSVVEKRLS....FMPAYRDLVITEQGKPFM..LG...ATATNIISLTENQ.......GVML
HsTET2 1318 .EEEKLESHLQ.NLSTLMAPTYKKLAP....DAYNNQIEYEHR.APECR..LGLKEGRPFSGVTACLDF.......CAHA
NgTET1 157 DLSQELGQYLSEIVNPQINYYISKLLTCVSSRTINYL.VSLN...DSYYALNN...CL.YPSTAFNSLKPSNDGHRIRKP
CMD1 345 HLDTDDG..VWTIILWFHRHS......GIIAGGEFVLPSLG..
HsTET2 1382 HRDLHNMQNGSTLVCTLTREDNREFGGKPEDEQLHVLPLYKVSDVDEFGSVEAQEEKKRSGAIQVLSSFRRKVRMLAEPV
NgTET1 229 HKDNLDI..TPSSLFYFGN.......FQNTEGYLELTDKN...................................
CMD1 378 ....................ISFQPLDFTIVVFAANTIVHGTRPLQTTGKI..IRWGSSHELRFKDVNALAQLGAAYG
HsTET2 1462 KTGSDEVWSDSEQSFLDPDIGGVAVAPTHGSILIECAKRELHATTPLKNPNRNHPTRISLVFYQHKSMNEPKHGL.....
NgTET1 260 ....................CKVFVQPGDVLFFKGNEYKHVVANITS....G..WRIGLVYEAHKGSKTK.PYYE....
CMD1 434 VDELDAKQRDQLEEVDAANSKDGVGAARRVASCMAAERKAA.IEAQKAA..CVRGVVMNPCTGRMPSLLFWQVWRKPP
HsTET2 1915 ....A.LWEAKMAEK...........................
NgTET1 308 ..........................DTQKNSLKIHKETK....................................

Supplementary Fig. 7. Structure-based sequence alignment of CMD1, HsTET2 and NgTET1. The alignment was performed with the Dali server [1]. Secondary structure elements of CMD1, HsTET2 and NgTET1 are placed on the top of the alignment. Strictly conserved residues are highlighted in shaded red boxes and conserved residues in open red boxes. The DSBH fold of the proteins is highlighted in yellow. The residues involved in the binding of the co-substrate and the recognition of the flipped-out 5mC of the DNA substrate in CMD1, HsTET2 and NgTET1 are highlighted in blue, orange and purple, respectively. The PDB codes of the structures used in the alignment are as follows: HsTET2 (4NM6) and NgTET1 (4LT5). The abbreviations for the species are as follows: Hs, *Homo sapiens*; Ng, *Naegleria gruberi*.

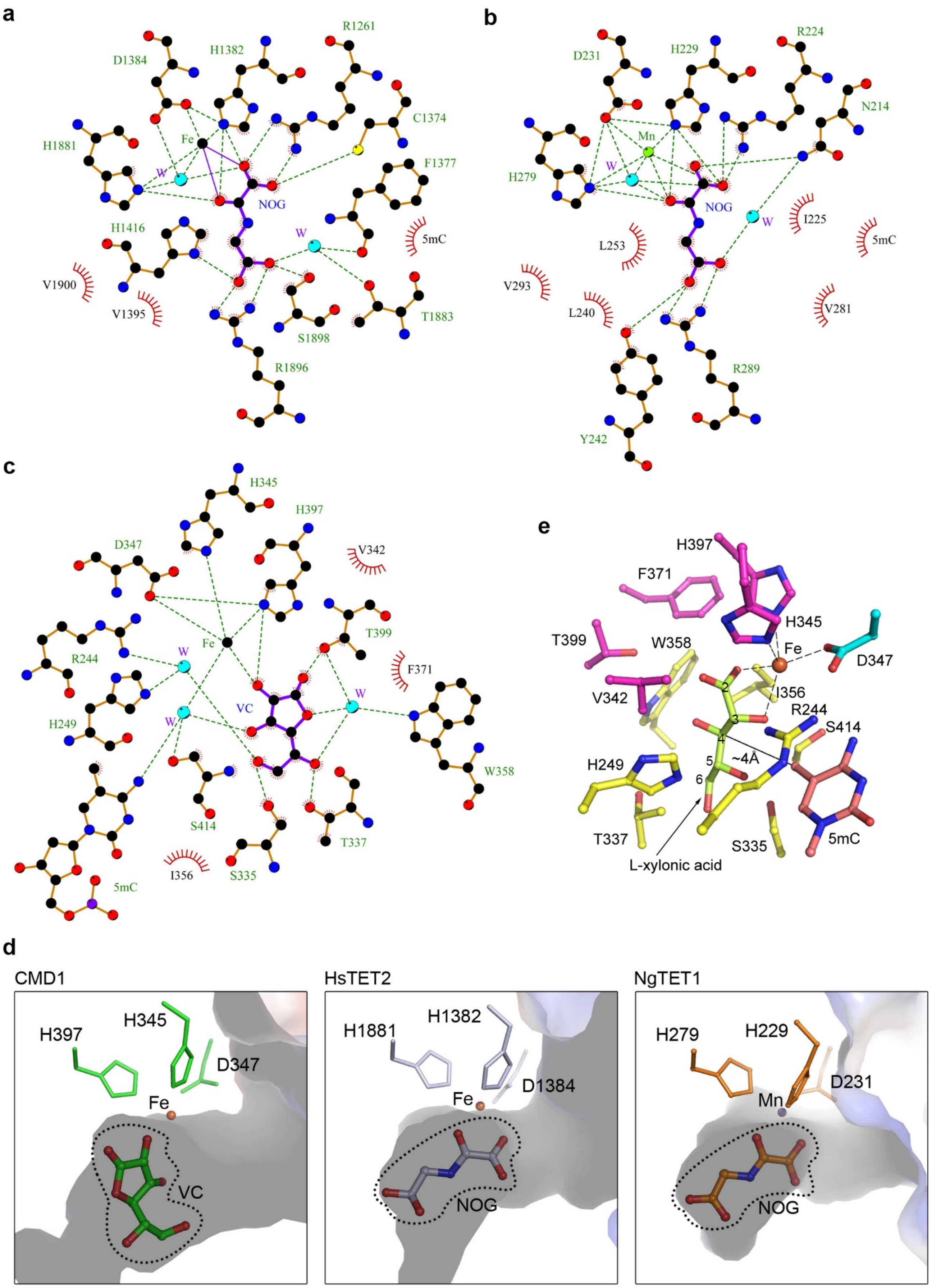
a
D1384
H1382
R1261
C1374
H1881
Fe
W
NOG
F1377
H1416
W
5mC
V1900
V1395
T1883
S1898
R1896
b
D231
H229
R224
N214
H279
Mn
W
NOG
W
I225
5mC
L253
V293
L240
V281
R289
Y242
c
H345
H397
D347
V342
T399
R244
Fe
W
F371
H249
W
VC
W
W358
S414
T337
5mC
I356
S335
e
H397
F371
H345
T399
W358
Fe
D347
V342
I356
R244
S414
H249
~4Å
T337
S335
5mC
L-xylonic acid
d
CMD1
H397
H345
D347
Fe
VC
HsTET2
H1881
H1382
D1384
Fe
NOG
NgTET1
H279
H229
D231
Mn
NOG

Supplementary Fig. 8. Structural comparison of CMD1 and the TET proteins. (a) Ligplot representation of the interactions between HsTET2 and NOG. (b) Ligplot representation of the interactions between NgTET1 and NOG. (c) Ligplot representation of the interactions between CMD1 and VC. The carbon, oxygen, and nitrogen are colored in black, red and blue, respectively. Hydrogen bonds are indicated with green dashed lines. Covalent bonds in protein and co-substrate are colored in brown and purple, respectively. (d) Comparison of the co-substrate binding pockets of CMD1, HsTET2 and NgTET1. The HXD…H motif and co-substrate or co-substrate analogue are shown with ball-and-stick models. The co-substrate binding pockets are shown with surface representation and circled with black dotted lines. (e) A modeling study of CMD1 with L-xylonic acid. The ligand is modeled based on the location of VC in the CMD1-5mC-DNA-VC complex. The distance between the C4 atom and the C5-methyl group of 5mC is indicated.

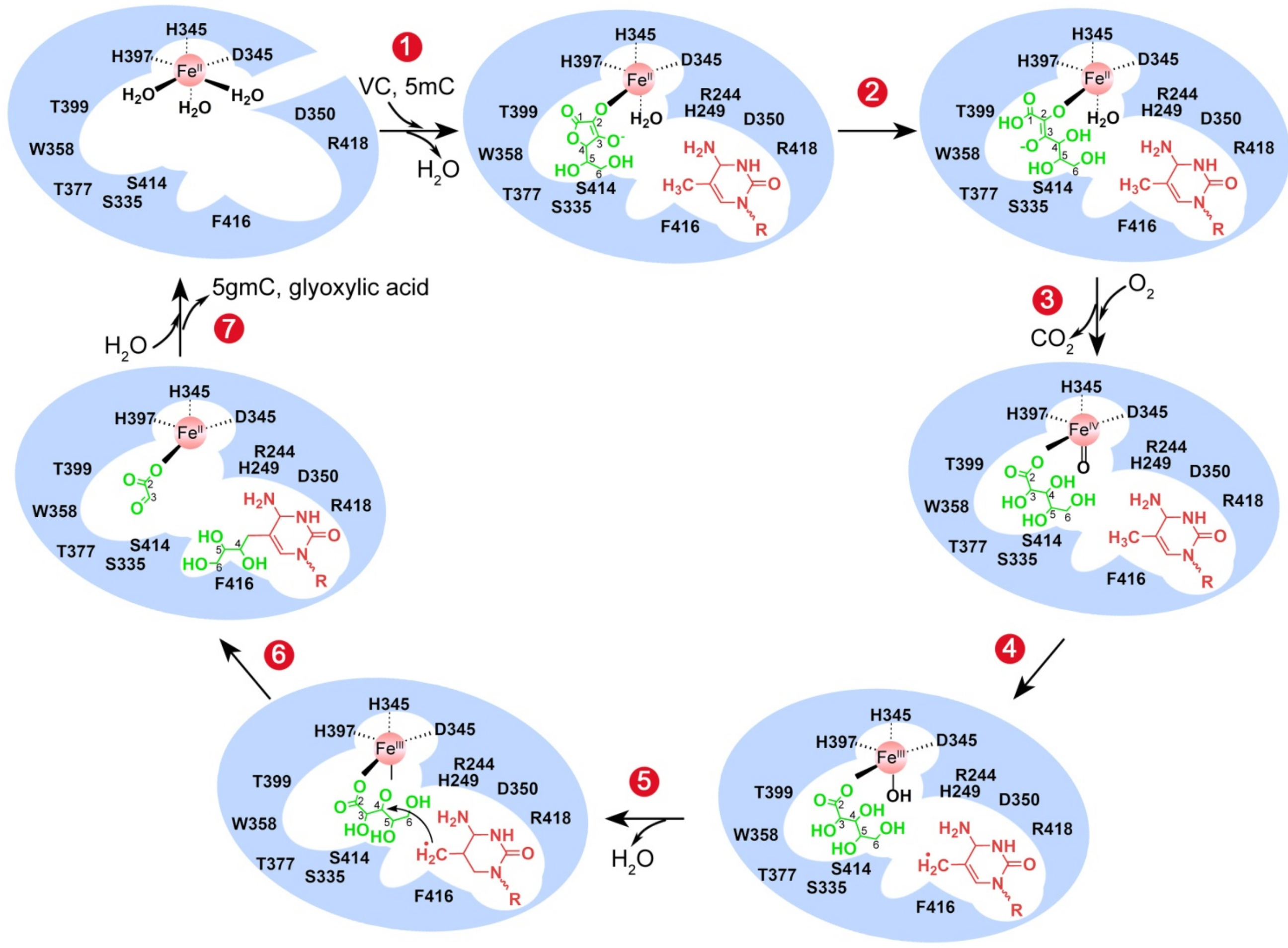

Supplementary Fig. 9. Proposed catalytic mechanism of CMD1. The proposed catalytic mechanism is modified based on the previously proposed catalytic mechanism of CMD1 [2]. Before the co-substrate and substrate bind to the active site of CMD1, the Fe^{2+} is coordinated by three strictly conserved residues (His345, Asp347 and His397) from the characteristic HXD/E…H motif and three water molecules with an octahedral geometry. At this stage, Arg244 and His249 from the β11-β12 loop are disordered. Then, the co-substrate and substrate bind to the active site of CMD1 in a non-sequential order and the lactone form of VC monocoordinates the Fe^{2+} via its C-2 hydroxyl group. Arg244 and His249 from the β11-β12 loop become ordered and are involved in the binding and recognition of 5mC-DNA (step 1). During the catalytic reaction, the ring-opened form of VC might maintain the monodentate coordination with the metal ion or undergo some conformational changes to coordinate the metal ion in a bidentate manner similar to 2-OG (step 2, the monodentate coordination manner is shown). Then, the ring-opened form of VC is decarboxylated to generate L-xylonic acid (or other intermediate) upon the activation of O_2 molecule by the metal ion using a similar mechanism as the other Fe^{2+}/2-OG-dependent dioxygenases (step 3). The C4-C6 moiety of

the ring-opened form or other intermediate of VC would change its conformation to swing towards the C5-methyl group of 5mC and might coordinate the metal ion in a bidentate mode similar to 2-OG (step 4-5). Finally, the C4 atom could be attacked by the 5mC radical to generate 5gmC and glyoxylic acid (step 6).

Supplementary References

1 Holm, L. & Rosenstrom, P. Dali server: conservation mapping in 3D. Nucleic Acids Res. 38, W545-549 (2010).

2 Xue, J. H. et al. A vitamin-C-derived DNA modification catalysed by an algal TET homologue. Nature 569, 581-585 (2019).
