# Supplementary material for: Molecular mechanism for vitamin C-derived C5-glyceryl-methylcytosine DNA modification catalyzed by algal TET homologue CMD1
Source: Nat Commun. 2021 Feb 2;12:744. doi: 10.1038/s41467-021-21061-2 (PMC7854593; doi:10.1038/s41467-021-21061-2)
Supplement: Supplementary file 3 — Reporting Summary [file 41467_2021_21061_MOESM3_ESM.pdf]

nature research

Corresponding author(s): Jianping Ding

Last updated by author(s): Dec 25, 2020

Reporting Summary

Nature Research wishes to improve the reproducibility of the work that we publish. This form provides structure for consistency and transparency in reporting. For further information on Nature Research policies, see our Editorial Policies and the Editorial Policy Checklist.

Statistics

For all statistical analyses, confirm that the following items are present in the figure legend, table legend, main text, or Methods section.

n/a	Confirmed	
☐	☒	The exact sample size (*n*) for each experimental group/condition, given as a discrete number and unit of measurement
☒	☐	A statement on whether measurements were taken from distinct samples or whether the same sample was measured repeatedly
☒	☐	The statistical test(s) used AND whether they are one- or two-sided<br>*Only common tests should be described solely by name; describe more complex techniques in the Methods section.*
☒	☐	A description of all covariates tested
☒	☐	A description of any assumptions or corrections, such as tests of normality and adjustment for multiple comparisons
☐	☒	A full description of the statistical parameters including central tendency (e.g. means) or other basic estimates (e.g. regression coefficient) AND variation (e.g. standard deviation) or associated estimates of uncertainty (e.g. confidence intervals)
☒	☐	For null hypothesis testing, the test statistic (e.g. *F*, *t*, *r*) with confidence intervals, effect sizes, degrees of freedom and *P* value noted<br>*Give P values as exact values whenever suitable.*
☒	☐	For Bayesian analysis, information on the choice of priors and Markov chain Monte Carlo settings
☒	☐	For hierarchical and complex designs, identification of the appropriate level for tests and full reporting of outcomes
☒	☐	Estimates of effect sizes (e.g. Cohen's *d*, Pearson's *r*), indicating how they were calculated

Our web collection on statistics for biologists contains articles on many of the points above.

Software and code

Policy information about availability of computer code

Data collection	X-ray diffraction data were collected at BL17U1 of Shanghai Synchrotron Radiation Facility or BL18U and BL19U1 of National Facility for Protein Science Shanghai. Blu-Ice 5.0 (beamline control software).
Data analysis	HKL2000, HKL3000 or XDS was used to process x-ray diffraction data. Single wavelength anomalous dispersion (SAD, implemented in Phenix 1.13) was used to solve the crystal structure of the Se-Met derivative CMD1. Phaser (implemented in Phenix 1.13) was used to solve the other structures using the apo CMD1 structure as the search model. Coot 0.8.2 was used to perform model building. Phenix 1.13 and Refmac 5 were used to perform structure refinement. CCP4 6.0.2 was used to carry out structural analysis. Pymol 2.0.7 was used to generate all the structure figures. Octet data analysis software 9.0 was used to analyze the binding curves of BLI assay. Qualitative Navigator was used to analyze the LC-MS results and integrate the 5gmC and 5mC peak. GraphPad Prism 8. 0. 2 was used to perform statistical analyses and generate the histogram of the enzymatic activity assay.

For manuscripts utilizing custom algorithms or software that are central to the research but not yet described in published literature, software must be made available to editors and reviewers. We strongly encourage code deposition in a community repository (e.g. GitHub). See the Nature Research guidelines for submitting code & software for further information.

Data

Policy information about availability of data

All manuscripts must include a data availability statement. This statement should provide the following information, where applicable:

- Accession codes, unique identifiers, or web links for publicly available datasets
- A list of figures that have associated raw data
- A description of any restrictions on data availability

Crystal structures have been deposited in the Protein Data Bank (PDB) with accession codes 7CY4 (CMD1 in apo form), 7CY5 (CMD1 in complex with vitamin C),

7CY6 (CMD1 in complex with 5mC-DNA), 7CY7 (CMD1 in complex with DNA) and 7CY8 (CMD1 in complex with both 5mC-DNA and vitamin C).

Field-specific reporting

Please select the one below that is the best fit for your research. If you are not sure, read the appropriate sections before making your selection.

☒ Life sciences ☐ Behavioural & social sciences ☐ Ecological, evolutionary & environmental sciences

For a reference copy of the document with all sections, see nature.com/documents/nr-reporting-summary-flat.pdf

Life sciences study design

All studies must disclose on these points even when the disclosure is negative.

Sample size	X-ray diffraction data were collected on single crystals until acceptable completeness and redundancy was reached. Sample-size choice was not predetermined by a statistical method. Three replicates were sufficient to show significant differences between different experimental groups.
Data exclusions	No data were excluded from the analysis.
Replication	The in vitro enzymatic activity experiment was successfully replicated with similar results for at least twice. All attempts at replication were successful. The bio-layer interferometry assay was performed twice with similar results and a representative experiment is presented in the paper. The co-substrate VC and/or substrate DNA bound at the active site of CMD1 were observed in more than five structural datasets. And the structures with highest resolution were used in detail analyses.
Randomization	5% of reflections for each dataset were randomly allocated by the PHENIX program for Rfree calculation.
Blinding	Because animal and clinical experiments and group analysis were not performed, blinding was not used in this study.

Reporting for specific materials, systems and methods

We require information from authors about some types of materials, experimental systems and methods used in many studies. Here, indicate whether each material, system or method listed is relevant to your study. If you are not sure if a list item applies to your research, read the appropriate section before selecting a response.

Materials & experimental systems

n/a	Involved in the study
☒	☐ Antibodies
☒	☐ Eukaryotic cell lines
☒	☐ Palaeontology and archaeology
☒	☐ Animals and other organisms
☒	☐ Human research participants
☒	☐ Clinical data
☒	☐ Dual use research of concern

Methods

n/a	Involved in the study
☒	☐ ChIP-seq
☒	☐ Flow cytometry
☒	☐ MRI-based neuroimaging
